# Beneficial influence of single-stage posterior surgery for the treatment of lumbar brucella spondylitis combined with spondylolisthesis

**DOI:** 10.1038/s41598-022-24223-4

**Published:** 2022-11-14

**Authors:** Yao Zhang, Changsong Zhao, Jiamin Chen, Qiang Zhang

**Affiliations:** 1 Department of Orthopedics, Beijing Ditan Hospital, Capital Medical University, No. 8, Jingshun East Street, Chaoyang District, Beijing, 100015 China; 2 Department of Pathology, Beijing Ditan Hospital, Capital Medical University, No.8, Jingshun East Street, Chaoyang District, Beijing, 100015 China

**Keywords:** Diseases, Medical research

## Abstract

We aimed to evaluate the clinical efficacy of the single-stage posterior surgical treatment for patients of lumbar brucella spondylitis combined with spondylolisthesis. In this study, we performed a retrospective analysis of 16 patients with lumbar brucellosis spondylitis combined with spondylolisthesis from January 2015 to January 2019. All patients underwent single-stage posterior lumbar debridement, reduction, interbody fusion, and instrumentation. Preoperative and postoperative of the visual analog scale (VAS), the Oswestry disability index (ODI), erythrocyte sedimentation rate (ESR), and C-reactive protein (CRP) were compared. In addition, the spondylolisthesis reduction rate, reduction loss rate, interbody fusion rate, and complication rate were recorded. VAS, ODI, ESR, and CRP were conducted with repeated analysis of variance data at different follow-ups. The postoperative follow-up was 12–36 months, with an average of (25.0 ± 8.1) months. VAS, ODI, ESR, and CRP were significantly better at 2-week and 1-year follow-up than preoperative results (*P* = 0.000, respectively). In addition, 1 year after the operation, VAS, ODI, ESR, and CRP showed a significant improvement (*P* = 0.000, respectively). The average spondylolisthesis reduction in 2 weeks after operation was (91.2 ± 6.7)%, and the median reduction loss rate in 1 year after operation was 8.0 (5.0, 9.8)%. At the last follow-up, all patients achieved interbody fusion, no loosening and fracture of instrumentation were found, and no recurrence happened. Single-stage posterior operation for lumbar debridement, reduction, interbody fusion, and instrumentation is beneficial for treating lumbar brucellosis spondylitis combined with spondylolisthesis. Furthermore, the reconstruction of spinal stability may relieve pain, heal lesions, and improve patients’ living.

## Introduction

Brucellosis is a common zoonosis affecting 500,000 new cases around the world annually. The disease is transmitted to humans through direct/indirect contact with infected animals or raw meat and dairy product consumption. In China, the brucellosis epidemic mainly exists in Inner Mongolia Autonomous Region and Hebei Province. Brucellosis could enter the human body through the respiratory tract, skin, and digestive tract, resulting in human fever and multiple organ damage^1,2^. Brucella mainly invades large joints, and the most frequently affected part is the spine^3^. From 2004 to 2018, a total of about 4,490,000 patients were diagnosed as brucellosis with an average annual incidence rate of 0.0259 per 100,000 population in China^4^. The incidence of brucella spondylitis is ranges from 2 to 53% in China^5,6^, which is prone to the lumbar spine. Due to the apparent destruction of the upper and lower edges of the vertebral body, it is easy to cause spinal instability^7^.

At present, antibiotic treatment combined with surgical treatment is still the primary method for treating Brucella spondylitis, and the clinical efficacy is not satisfactory for patients with antibiotic treatment only. The patients with brucella spondylitis require surgical treatment, accounting for 3–29%^5^. After spinal infection with Brucella, necrotic tissue or intervertebral disc destruction occurs in the spinal canal. These lesions will cause indirect or direct compression of the nerve and neurological symptoms. In addition, antibiotic treatment can not relieve the symptoms caused by the compression of the spinal cord, cauda equina nerve, and nerve root, so surgical treatment is required^8^.

Lumbar spondylolisthesis is one of the common causes of low back and leg pain, and the specific pathogenesis is unclear. Its classification and treatment methods are diverse. The treatment principle is to restore the spinal sequence as much as possible and eliminate the causes of pain^9^. At present, there is no unified theory about the choice of conservative and surgical treatment, the choice of surgical approach, the choice of minimally invasive and open, and the choice of fusion and decompression^10^.

Currently, the surgical indications for brucella spondylitis are not unified worldwide. Over the past years, as far as brucella spondylitis is concerned, antibiotic treatment has been deemed the primary therapeutic strategy. However, surgical treatment is warranted for patients who still experience persistent low back pain, progressive neurological functional dysfunction, and spinal instability after antibiotic treatment has been administered. So far, there are few reports on surgical treatment of lumbar brucella spondylitis combined with spondylolisthesis. This study evaluated the clinical efficacy of the single-stage posterior surgical treatment for lumbar brucella spondylitis combined with spondylolisthesis.

## Materials and methods

### Patient population

**Figure 1:**
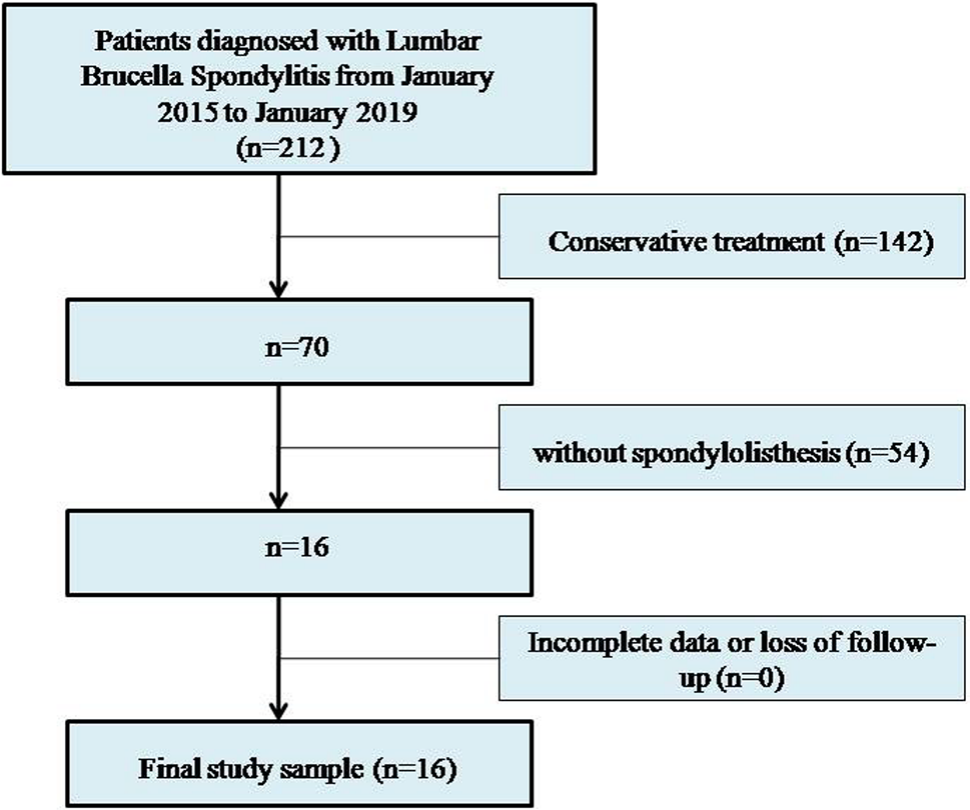
Flowchart showing study enrollment.

From January 2015 to January 2019, we included 212 patients with lumbar brucella spondylitis in our department. All the patients were initially treated with antibiotic treatment, 142 patients had relief after antibiotic treatment (no one combined with spondylolisthesis), and the other 70 patients had poor recovery and met the indications for surgery. Patients met the following indications for surgery: (1) Persisting low back pain; (2) Progressive neurological deficit; (3) Spinal instability: abnormal movement between one vertebra and another caused by the destruction of facet joints, et cetera; (4) Paravertebral abscess or epidural abscess not easily absorbed; (5) Poor outcomes following antibiotic treatment. 16 cases of lumbar brucella spondylitis combined with spondylolisthesis underwent single-stage posterior debridement, reduction, interbody fusion, and instrumentation by the same surgeon Professor Zhang who has rich experience in diagnosing and treating brucellosis spondylitis (Fig. 1).

The inclusion criteria were as follows: (1) Epidemic history: living in or having been to pastoral areas, having exposure to cattle and sheep, or eating non sterilized cattle, mutton, or dairy products; (2) Symptoms and signs: fever, sweats, fatigue, weight loss, low back pain, and lower limb nerve symptoms; (3) Imaging findings: preoperative X-ray, CT, and MRI findings were consistent with the signs of brucella spondylitis and combined with spondylolisthesis; (4) Serological and microbiological evidence: Rose Bengal plate agglutination test (RBP) was positive or histopathological examination was positive; (5) No other etiological findings (such as *Mycobacterium* *tuberculosis*, *Staphylococcus aureus*, fungi, etc.).

This retrospective study was approved by the ethical committee and performed in accordance with the ethical standards of the 1964 Declaration of Helsinki. All patients in this study voluntarily signed an informed consent form to join the scientific research and signed an informed consent form for surgical treatment.

### Data collection

The operation time, intraoperative bleeding, and complications were recorded. The patients were followed up regularly at 2 weeks, 1 month, 2 months, 3 months, 6 months, and 1 year after the operation. During the follow-up, X-rays of the lumbar spine were performed in the frontal, lateral and dynamic positions, and a CT scan of the lumbar spine would be performed if necessary. We calculated the spondylolisthesis reduction rate 2 weeks after operation by measuring the vertebral body slippage, i.e. (preoperative slippage − postoperative slippage)/preoperative slippage × 100%^11^. The spondylolisthesis rate at 2 weeks and 1 year after operation was measured by Taillard's measurement^12^, and the reduction loss rate was also calculated. In addition, we observed the condition of the fusion of bone graft and checked the instrumentation 2 weeks and 1 year after the operation^13,14^. The visual analog scale (VAS), the Oswestry disability index (ODI), and laboratory tests included preoperative and postoperative erythrocyte sedimentation rate (ESR), and C-reactive protein (CRP) were recorded. Meantime, the neurological status was evaluated according to the preoperative and postoperative American Spinal Injury Association (ASIA) scale.

### Preoperative preparation

Patients were treated with combinations of antibiotics. The regimens included oral doxycycline 200 mg/day, oral rifampicin 600 mg/day, intravenous levofloxacin 500 mg/day, or intravenous ceftriaxone sodium 2 g/day for at least 6 weeks. In addition, nutrition enhancement and correction of anemia and hypoproteinemia in needed patients were also carried out. The same antibiotic regimen was continued for at least 2 weeks after surgery in all patients, followed by oral treatment of doxycycline and rifampicin for 6 months.

### Surgical techniques

**Figure 2:**
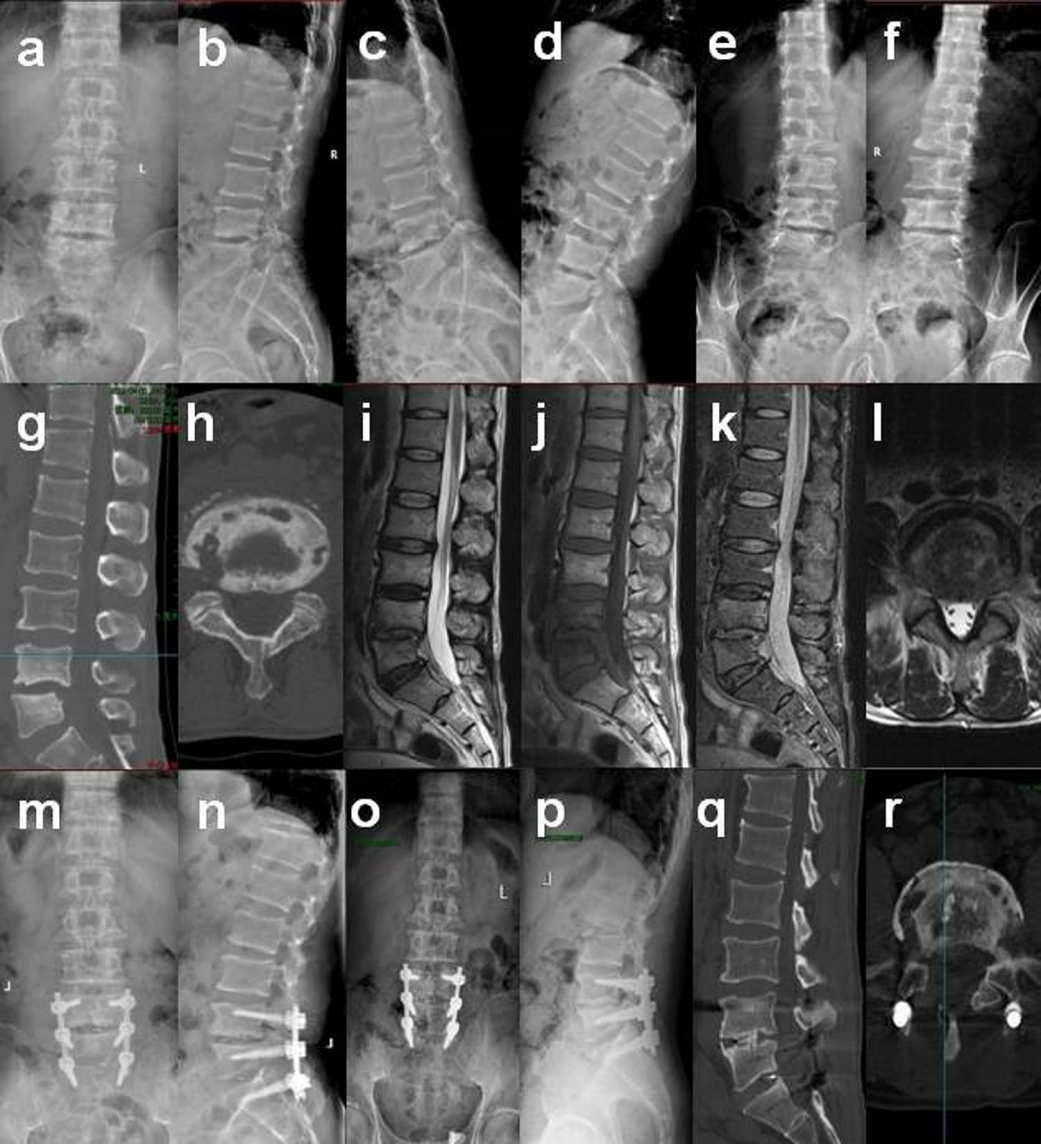
Preoperative and postoperative imaging findings of typical patients. (**a**–**f**) Preoperative X-ray showed L4 vertebral body posterior slippage and lumbar instability in a dynamic position, and no obvious isthmus was found in a double oblique position. (**g**,**h**) Preoperative CT showed L4–5, L5–S1 multiple lesions at the upper and lower edges of the vertebral body, especially L4–5. (**i**–**l**) Preoperative MRI showed L4–5 and L5–S1 uneven abnormal signal changes in the vertebral body and intervertebral disc. (**m**,**n**) Two weeks after the operation, X-ray showed L4 spondylolisthesis reduction. (**o**,**p**) One year after the operation, the X-ray showed no apparent loss of spondylolisthesis reduction, and the spine was stable. (**q**,**r**) One year after the operation, CT showed interbody fusion had been achieved, and the damaged area was repaired well.

Under general anesthesia, the patients were placed in the prone position. The pedicle of the spondylolisthesis vertebral body was determined and marked fluoroscopically. First, the posterior median incision was made. Then, the skin, subcutaneous, and fascia layers were incised successively and separated to both sides along the spinous process until the lamina and facet joints. Then reduction screws were placed on both sides of the spondylolisthesis vertebral body, and ordinary pedicle screws were placed in the adjacent vertebral body. Subsequently, unilateral or bilateral fenestration decompression was performed. Then, the inflammatory tissues in the spinal canal, intervertebral space, paravertebral and damaged bone were removed entirely, and the intervertebral space was removed until there was blood exudation. Then, we repeatedly washed it with a flushing gun, dried it, installed the longitudinal connecting rod, and lifted and restored the spondylolisthesis vertebral body while opening the intervertebral space properly. Finally, the previously obtained bone granule and/or allograft bone were implanted into the bone defect between vertebrae, and then the polyetheretherketone (PEEK) cage was obliquely inserted. After moderate compression, it was tightened and fixed. Then, we rewashed the gun. After checking that there was no active bleeding, we placed a drainage tube routinely and sutured the incision layer by layer. In the end, the tissues taken during the operation were sent for pathological examination (Fig. 2).

### Statistical analysis

All statistical analyses were performed using the SPSS software (version 25.0; IBM SPSS, New York, USA). Continuous variables were presented as mean ± standard deviation or medians with interquartile ranges, while categorical variables as the frequencies or percentages of events. Mann–Whitney *U* test was used for nonnormally distributed continuous variables and a *t* test for normally distributed variables. VAS, ODI, ESR, and CRP were conducted with repeated measures analysis of variance data. The *P* value ≤ 0.05 was considered to indicate statistical significance.

### Ethics approval and consent to participate

This retrospective study was approved by the ethical committee of Beijing Ditan Hospital, Capital Medical University and performed in accordance with the ethical standards of the 1964 Declaration of Helsinki. And all participants signed informed consent for publication.

## Results

### Basic information

Among the 16 patients, there were 14 males and 2 females. The age ranged from 46 to 68 years, with an average of (59.2 ± 6.5) years. The follow-up time of 16 patients ranged from 12 to 36 months, with an average of (25.0 ± 8.1) months. In addition, 12 patients had a history of living in or having been to pastoral areas, and 14 patients had a history of having exposure to cattle and sheep or eating non sterilized cattle, mutton, or dairy products.

All patients had back pain (100%), followed by weakness or fatigue (87.5%), fever (75%), sweats (75%), then weight loss and neurological symptoms of lower limbs (62.5%, respectively).

There were 3 cases of L1/2 slippage, 2 cases of L2/3 slippage, 1 case of L3/4 slippage, 4 cases of L4/5 slippage, and 6 cases of L5/S1 slippage. There were 9 cases of anterior slippage and 7 cases of posterior slippage, including 10 cases with the isthmus. Meantime, there were 12 cases of vertebral body destruction, 6 cases of epidural abscess, and 2 cases of paravertebral abscess (Table 1).

**Table 1:** Clinical data of patients.

	N (%)		N (%)
**Epidemic history**		**Radiographic features**	
Living in or having been to pastoral areas	12 (75%)	Vertebral body destruction	12 (75%)
Contact history	14 (87.5%)	Epidural abscess	6 (37.5%)
**Clinical symptoms**		Paravertebral abscess	2 (12.5%)
Fever	12 (75%)	Isthmus	10 (62.5%)
Sweats	12 (75%)	**Spondylolisthesis segment**	
Weakness or fatigue	14 (87.5%)	L1/2	3 (18.8%)
Weight loss	10 (62.5%)	L2/3	2 (12.5%)
Back pain	16 (100%)	L3/4	1 (6.3%)
Neurological symptoms of lower limbs	10 (62.5%)	L4/5	4 (25%)
**Serological evidence and microbiological evidence**		L5/S1	6 (37.5%)
Rose Bengal plate agglutination test	16 (100%)	Anterior slippage	9 (56.3%)
Histopathological examination	16 (100%)	Posterior slippage	7 (43.8%)
**Extent of surgery**		**Other pathogens**	0
One level	13 (81.2%)	–	–
Two levels	3 (18.8%)	–	–
Three levels	0	–	–

### Surgical data and findings

The operation time of 16 patients ranged from 1.5 to 3.0 h, the median was 2.0 (1.5, 2.0) h, the amount of bleeding was 300–550 ml, and the average was (403.1 ± 64.2) ml. There were 13 cases fused in one level and 3 cases fused in two levels (Table 1). The incisions of 14 patients healed in the first stage, and 2 patients healed delayed due to local scabs. There were no related complications, and no spinal cord, cauda equina, nerve root, and vascular injury were found early after the operation.

**Figure 3:**
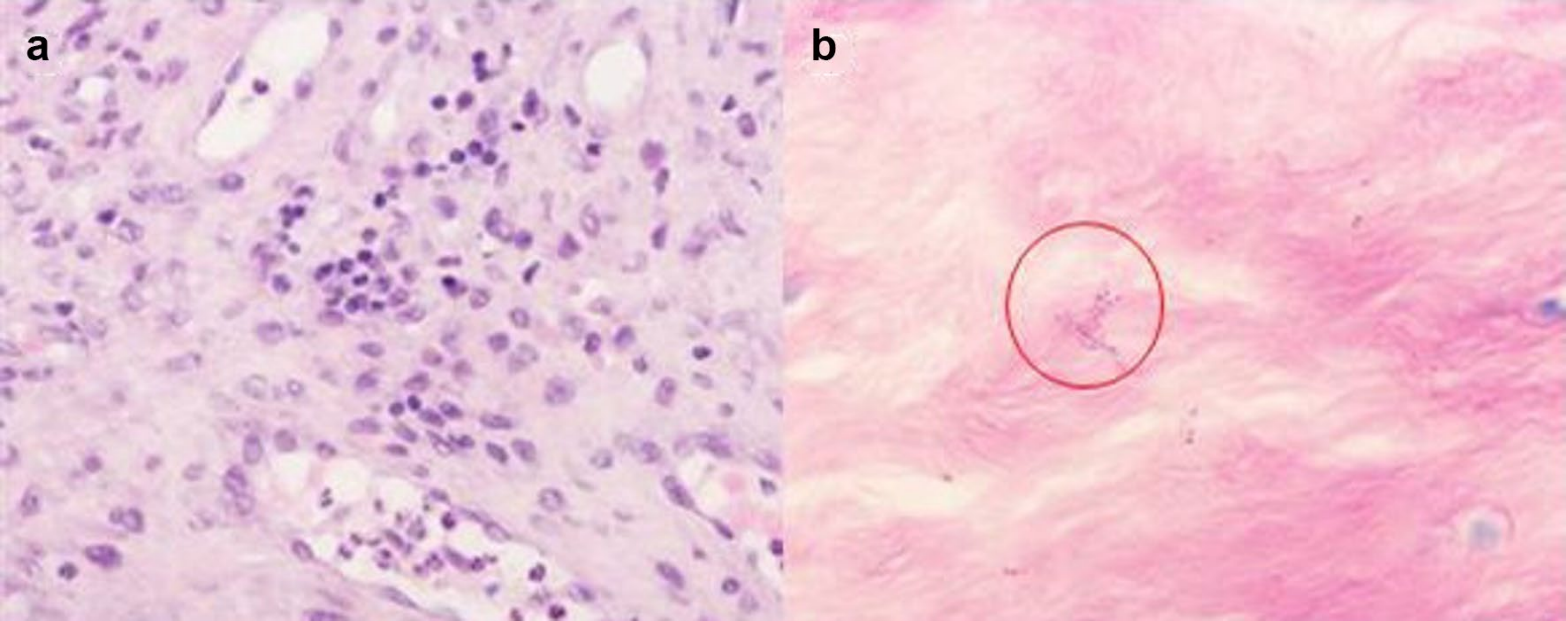
Histopathological results of postoperative lesions. (**a**) HE staining showed many different types of inflammatory cells in the lesions (× 400). (**b**) Brucella saw by Giemsa staining (× 1000).

The pathological HE staining of 16 patients showed many inflammatory cells such as monocytes, lymphocytes, and neutrophils in the lesion area, which was consistent with the changes in brucellosis. Brucella could be seen by Giemsa staining (Fig. 3).

### Comparison of preoperative and postoperative lumbar function index

The VAS score decreased from 8.0 (8.0, 8.8) preoperatively to 2.0 (1.3, 2.0) and 0.0 (0.0, 1.0) at 2 weeks and 1 year after the operation, respectively. ODI index decreased from (88.5 ± 5.6)% preoperatively to (35.7 ± 3.1)% and (9.3 ± 5.7)% at 2 weeks and 1 year after the operation, respectively. VAS score, and ODI index at 2 weeks and 1 year after the operation were significantly different from those before the operation (*P* = 0.000, Table 2). Moreover, 1 year after the operation, the VAS score, and ODI index were significantly different from those 2 weeks after the operation (*P* = 0.000, Table 2). The ASIA scale of 16 patients before operation is as follows: 1 case was grade B, 2 cases were grade C, 7 cases were grade D, and 6 cases were grade E. 1 year after the operation, the neurological function of all 10 patients recovered after surgery, and the ASIA scale improved by 1–2 grades.

**Table 2:** Comparison of preoperative and postoperative VAS, ODI, ESR, and CRP.

	VAS	ODI (%)	ESR (mm/h)	CRP (mg/L)
Preoperative	8.0 (8.0, 8.8)	88.5 ± 5.6	35.5 (14.5, 43.0)	20.3 ± 10.2
2-Week follow-up	2.0 (1.3, 2.0)	35.7 ± 3.1	12.9 ± 5.3	7.6 ± 3.1
1-Year follow-up	0.0 (0.0, 1.0)	9.3 ± 5.7	9.2 ± 3.6	3.5 ± 1.7
Z/T	–	612.140	–	37.095
*P*	0.000	0.000	0.000	0.000

VAS: preoperative vs. 2-week follow-up: Z = − 3.551, *P* = 0.000; preoperative vs. 1-year follow-up: Z = − 3.543, *P* = 0.000; 2-week follow-up vs. 1-year follow-up: Z = − 3.508, *P* = 0.000.

ODI: preoperative vs. 2-week follow-up: T = 31.327, *P* = 0.000; preoperative vs. 1-year follow-up: T = 36.063, *P* = 0.000; 2-week follow-up vs. 1-year follow-up: T = 20.702, *P* = 0.000.

ESR: preoperative vs. 2-week follow-up: Z = − 3.519, *P* = 0.000; preoperative vs. 1-year follow-up: Z = − 3.411, *P* = 0.001; 2-week follow-up vs. 1-year follow-up: t = 2.577, *P* = 0.021.

CRP: preoperative vs. 2-week follow-up: t = 5.025, *P* = 0.000; preoperative vs. 1-year follow-up: t = 6.688, *P* = 0.000; 2-week follow-up vs. 1-year follow-up: t = 6.619, *P* = 0.000.

### Comparison of preoperative and postoperative ESR and CRP

ESR and CRP gradually decreased to normal in 16 patients. ESR decreased from 35.5 (14.5, 43.0) mm/h preoperatively to (12.9 ± 5.3) mm/h and (9.2 ± 3.6) mm/h at 2 weeks and 1 year after the operation, respectively. CRP decreased from (20.3 ± 10.2) mg/L preoperatively to (7.6 ± 3.1) mg/L and (3.5 ± 1.7) mg/L at 2 weeks and 1 year after the operation, respectively. ESR and CRP at 2 weeks and 1 year after the operation were significantly different from those before the operation (*P* = 0.000, Table 2). Moreover, 1 year after the operation, ESR and CRP were significantly different from those 2 weeks (*P* = 0.000, Table 2).

### Radiological evaluation

The average spondylolisthesis reduction rate 2 weeks after operation was (91.2 ± 6.7)%, and the median reduction loss rate 1 year after operation was 8.0 (5.0, 9.8)%. At the last follow-up, all patients achieved interbody fusion without loosening or fracture of instrumentation, and no patient had recurrence (Fig. 2).

## Discussion

The pathological changes of brucella spondylitis are mainly infectious vertebrates and discitis, with vertebral destruction, spinal instability, and spinal cord or nerve compression^3–6,15^. In recent years, with further research on spinal biomechanics, researchers believed that surgery should be carried out to remove the lesions and reconstruct spinal stability to relieve pain and promote the recovery of nerve function when antibiotic treatment is not enough for curing^8,16,17^. Tebet^18^ classified the causes of spondylolisthesis as isthmic, degenerative, and traumatic, especially isthmus and degeneration. However, few studies have been done on the correlation between brucella spondylitis and lumbar spondylolisthesis. There is also a lack of statistical data on the incidence of lumbar spondylolisthesis in patients with brucella spondylitis. As we all know, the spine's stability depends on the joint maintenance of the complete vertebral body, intervertebral disc, pedicle, ligament, and surrounding tissue. In this study, 10 of the 16 patients (62.6%) were combined with isthmus, with a high proportion, which may be one of the factors of lumbar spondylolisthesis. In further research, we may be able to perform correlation studies.

Clinically, various surgical methods for brucella spondylitis include anterior, posterior, or anterior and posterior debridement for bone graft fusion, and minimally invasive surgical methods, which emerged in recent years^8,16,17,19,20^. The traditional surgical methods of brucella spondylitis are anterior and posterior combined debridement, bone graft fusion, and instrumentation. This method has obvious disadvantages, including long operation time, changing body position during operation, and too complex operation^21,22^. Therefore, more surgeons have chosen posterior debridement, bone graft fusion, and instrumentation in recent years. This operation avoids the shortcomings of traditional operations and can significantly reduce the probability of recurrence^16,17,19^. Na et al.^16^ showed that the posterior approach gives better kyphotic deformity correction, less surgical invasiveness, and fewer complications in patients combined with lumbar brucella spondylitis. Zhao et al.^17^ showed that using polyetheretherketone (PEEK) cages combined with one-stage posterior debridement and instrumentation is feasible and safe in patients suffering from lumbar brucella spondylitis. Chen et al.^19^ showed that one-stage debridement, autogenous bone graft, and instrumentation via a posterior approach could represent an alternative treatment for lumbar brucella spondylitis, and the efficacy and safety of these techniques are satisfactory. The surgical methods of lumbar spondylolisthesis are also diverse. The most classic surgical methods are posterior interbody fusion. Transforaminal and extreme lateral approaches gradually developed, including the oblique lateral approach, which has sprung up in recent years, are posterior. The surgical core is reduction, decompression, fixation, and fusion^23,24^. So, we adopted a posterior approach in this study.

The spine's stability needs a typical spinal sequence to maintain, but there are still disputes about whether the spondylolisthesis needs reduction and the degree of reduction. Poussa et al.^25^ showed that in situ fusion of spondylolisthesis vertebral body could also achieve satisfactory clinical results, while spondylolisthesis reduction might bring discomfort for patients. On the other hand, Dewald et al.^26^ reported that complete reduction would lead to excessive nerve root traction. Therefore, a partial reduction of the spondylolisthesis vertebral body should be recommended. More researchers^27,28^ suggested reducing the spondylolisthesis vertebral body to the greatest extent, which will help restore the typical spinal sequence and promote bone graft fusion. As the postoperative recovery of brucella spondylitis needs stability, we reduced the spondylolisthesis vertebral body during the operation as much as possible in this study.

However, the reduction is temporary, and bone graft fusion is the ultimate goal. The use of internal fixation instruments could maintain the reduction of the spondylolisthesis vertebral body, and bone graft fusion would help the stability of the spine^29^. Dantas et al.^30^ found that posterolateral bone graft was not on the load-bearing axis, which was prone to the non-fusion of bone graft or formation of the pseudo joint. Through a follow-up study, Miyashita et al.^31^ found that pedicle screws and interbody fusion cage could rebuild spinal stability, effectively maintain the height of intervertebral space, reduce screw pressure, and reduce the incidence of fracture and loosening of pedicle screws. Compared with simple debridement and bone graft fusion, instrumentation could make the local stability conducive to bone graft fusion and promote lesions cure, with fewer postoperative complications and a low recurrence rate^8,17,32^. Bone graft fusion is the basis of spinal stability. Therefore, internal fixation instruments are conducive to bone graft fusion. Said et al.^33^ researched that the patients with lumbar spondylolisthesis combined with an isthmus, posterior interbody fusion might restore spinal stability, improve bone graft fusion, and have an excellent long-term clinical effect. In this study, 62.6% of patients were complicated with an isthmus. Therefore, we adopted polyetheretherketone (PEEK) cages, interbody fusion, and pedicle screw fixation.

The postoperative follow-up showed that the average spondylolisthesis reduction rate in 16 patients was (91.2 ± 6.7)% 2 weeks after the operation. The median reduction loss rate was 8.0 (5.0, 9.8)% 1 year after the operation, indicating the beneficial influence of the surgery. At the last follow-up, all patients had an interbody fusion, no loosening, fractures of instrumentation were found, and no patient had a recurrence, which means satisfactory clinical outcomes. During the follow-up, the clinical symptoms of 16 patients gradually improved, and the inflammatory indexes were back to normal. In addition, we found that the VAS score, ODI index, ESR, and CRP at 2-week and 1-year follow-up were significantly different from those before the operation, which showed a marked improvement in the patient's condition. Moreover, VAS score, ODI index, ESR, and CRP at 1-year follow-up were significantly better than those at 2-week follow.

Our study had limitations: First, the number of patients with lumbar brucellosis spondylitis combined with lumbar spondylolisthesis was small, and there was a lack of relevant research. Second, in this study, among the 142 patients with lumbar brucellosis spondylitis who improved after antibiotic treatment, none of them were combined with lumbar spondylolisthesis, so a control analysis could not be carried out, which related to the limited period of our retrospective analysis and the insufficient number of patients. Third, the follow-up time was short. Through our previous observation, most patients returned to normal after 1 year of the operation, so we have done a 1-year follow-up in this research. In future work, we will extend the follow-up time and conduct a statistical analysis of relevant data. Therefore, further observation, research, and clinical evaluation of cohort studies are needed.

## Conclusion

In conclusion, a single-stage posterior approach could achieve a beneficial outcome for patients with lumbar brucella spondylitis combined with spondylolisthesis. Posterior lumbar debridement, reduction, interbody fusion, and instrumentation could reconstruct the spine's stability, relieve pain and improve the patients' life quality.

## Data Availability

The datasets used and analyzed during the current study are available from the corresponding author upon reasonable request.

## Abbreviations

VAS: The visual analog scale
ODI: The Oswestry disability index
ESR: Erythrocyte sedimentation rate
CRP: C-reactive protein
CT: Computed tomography
MRI: Magnetic resonance imaging
